# Distinguishing direct androgenic signaling from local aromatization in the lateral septum

**DOI:** 10.1073/pnas.2618568123

**Published:** 2026-07-06

**Authors:** Dong’e Huang, Junqing Dong

**Affiliations:** ^a^Department of Traditional Chinese Medicine, The 900th Hospital of the Joint Logistics Support Force, Fuzhou, Fujian 350004, China; ^b^Department of Physiotherapy, 900th Hospital of the Joint Logistics Support Force, Ningde, Fujian 352103, China

We read with great interest the recent study by Aspesi et al., which proposes a bidirectional lateral septum (LS) circuit governing social cognition via androgens and estrogens (1). While the behavioral phenotyping is rigorous, we identify three critical mechanistic ambiguities that weaken the claim of direct, nonaromatizable androgen–estrogen receptor (ER) crosstalk in the LS.

First, the attribution of testosterone (T) effects to direct ER binding overlooks the well-documented, high-density aromatase expression in the LS and adjacent limbic structures (2, 3). Without concomitant use of aromatase inhibitors (e.g., letrozole) or aromatase-knockout (ArKO) models, the observed behavioral and neurochemical shifts cannot be decisively separated from rapid, local T-to-estradiol (E_2_) conversion (4). Second, the neurochemical validation relies on a methodological discontinuity: Immunohistochemistry demonstrates enhanced arginine vasopressin (AVP) fiber density following T or DHT treatment, whereas microdialysis indicates extracellular AVP release exclusively with E_2_ administration. This conflates peptide storage/transport with dynamic synaptic release, a known pitfall in neuropeptidergic signaling studies (5). Third, receptor knockdown alone cannot dissociate genomic from nongenomic (membrane-initiated) steroid signaling. T and E_2_ both rapidly modulate LS neuronal excitability via membrane-associated receptors, a pathway not addressed by the current genetic approach.

To robustly establish a direct androgenic pathway, we strongly recommend: i) quantifying local aromatase activity and incorporating pharmacological blockade to isolate T-specific effects; ii) employing in vivo fiber photometry or optogenetic-AVP reporters to align peptide dynamics with real-time behavioral states; and iii) complementing genetic knockdowns with rapid membrane-receptor antagonists (e.g., G15 for GPER). Resolving these confounds will substantially clarify whether androgens directly modulate LS social circuits or act primarily via classical neurosteroidogenesis.

## Data Availability

Previously published data were used for this work (1). All other data are included in the article.
